# The Retrospect of Practical Medicine and Surgery for the Year 1840; Being an Analysis of the British and Foreign Medical Journals and Transactions, or a Selection of the Latest Discoveries and Most Practical Observations in the Practice of Medicine, Surgery, and the Collateral Sciences for the past Year; Made Chiefly with References to the Treatment of Disease

**Published:** 1841-01

**Authors:** 


ART. X.—*The Retrospect of Practical Medicine and Surgery for the year* 1840; *being an Analysis of the British and Foreign Medical Journals and Transactions, or a Selection of the latest Discoveries and most Practical Observations in the practice of Medicine, Surgery, and the collateral Sciences for the past year: made chiefly with reference to the Treatment of Disease.* By W. BRAITHWAITE, M.R.C.S.—*London,* 1840. 12mo, pp. 208.

THE nature of this little work is accurately indicated in the above title; we will only add that the plan is extremely well carried out in the present number, and that the undertaking merits the patronage of the profession. We hope Mr. Braithwaite will meet with such support as to induce him to continue his labours.

